# Supplementary material for: Long-acting injectable atovaquone nanomedicines for malaria prophylaxis
Source: Nat Commun. 2018 Jan 22;9:315. doi: 10.1038/s41467-017-02603-z (PMC5778127; doi:10.1038/s41467-017-02603-z)
Supplement: Supplementary file 1 — Supplementary Information [file 41467_2017_2603_MOESM1_ESM.pdf]

Supplementary Information

Supplementary Figures

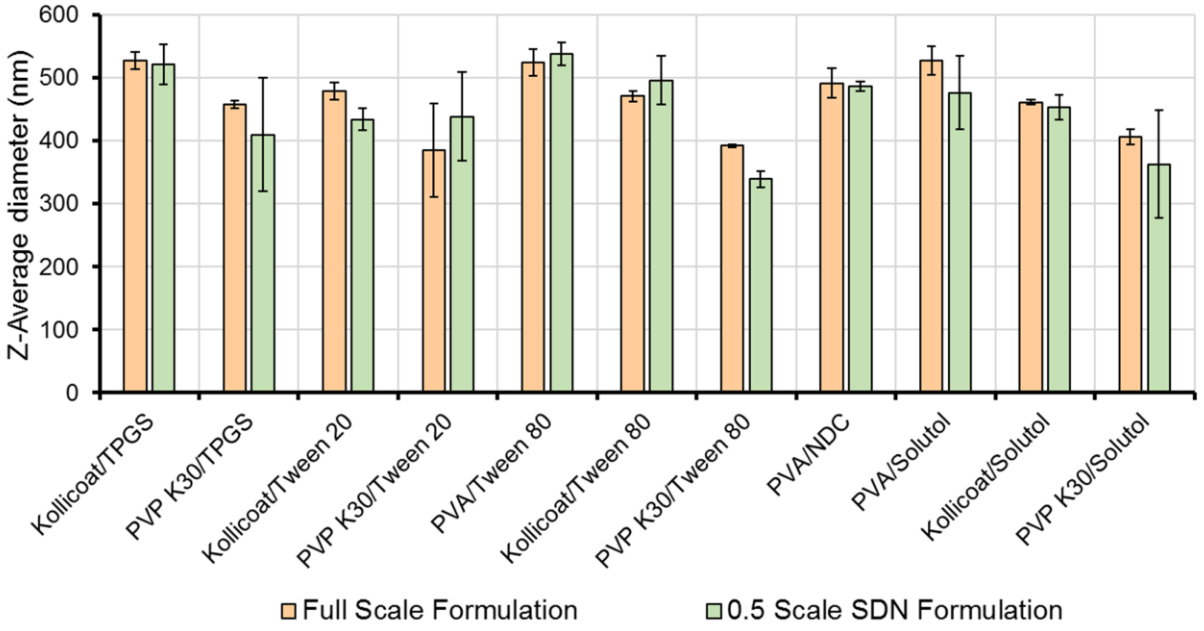

Supplementary Fig. 1 Characterization of nanoformulation candidates. Formulation reproducibility of screen 'hits' (error bars denote ±1 SD; three repeats) with concordance between full-scale and half-scale ETFD; ^{3}H-labelled atovaquone inclusion used on half-scale SDN synthesis.

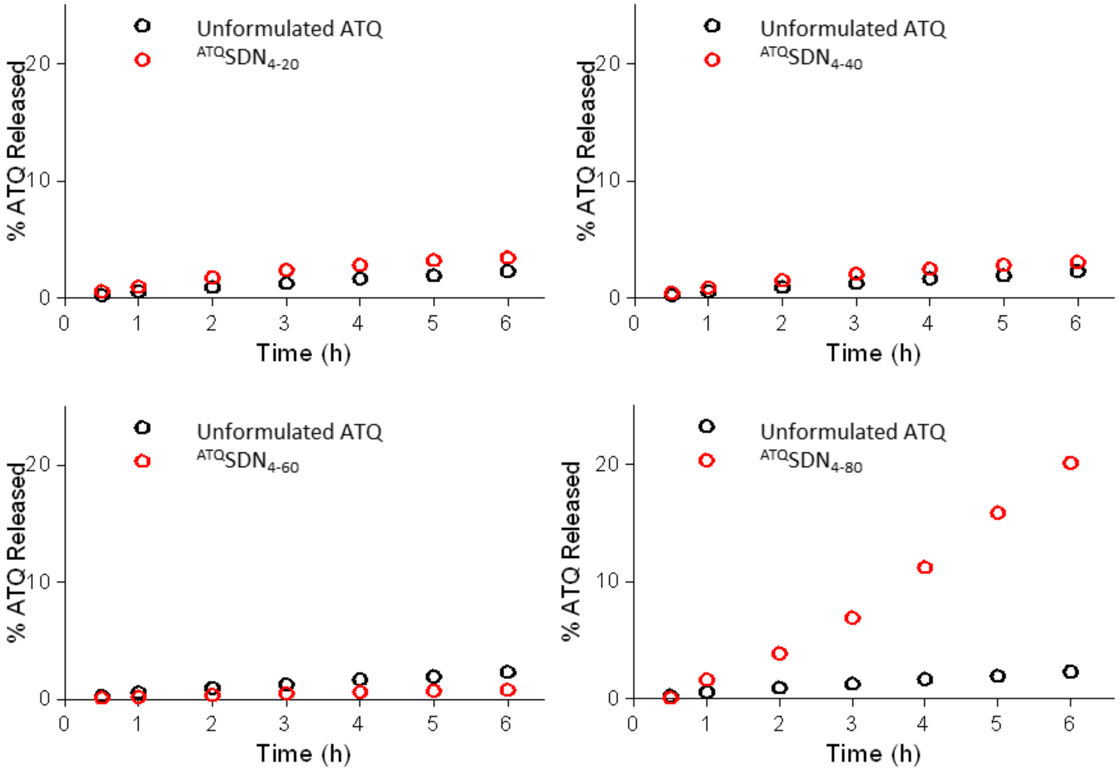

Supplementary Fig. 2 Comparison of rapid equilibrium dialysis release rate measurements of $^{ATQ}SDN_4$ at 20 ($^{ATQ}SDN_{4\text{-}20}$), 40 ($^{ATQ}SDN_{4\text{-}40}$), 60 ($^{ATQ}SDN_{4\text{-}60}$) and 80 ($^{ATQ}SDN_{4\text{-}80}$) wt% drug loading. For release rate studies, samples were prepared incorporating ^{3}H-atovaquone at 0.2 μCi mg^{-1} specific activity. $^{ATQ}SDN_4$ was selected on the basis that it exhibited the highest release rate in the initial screen.

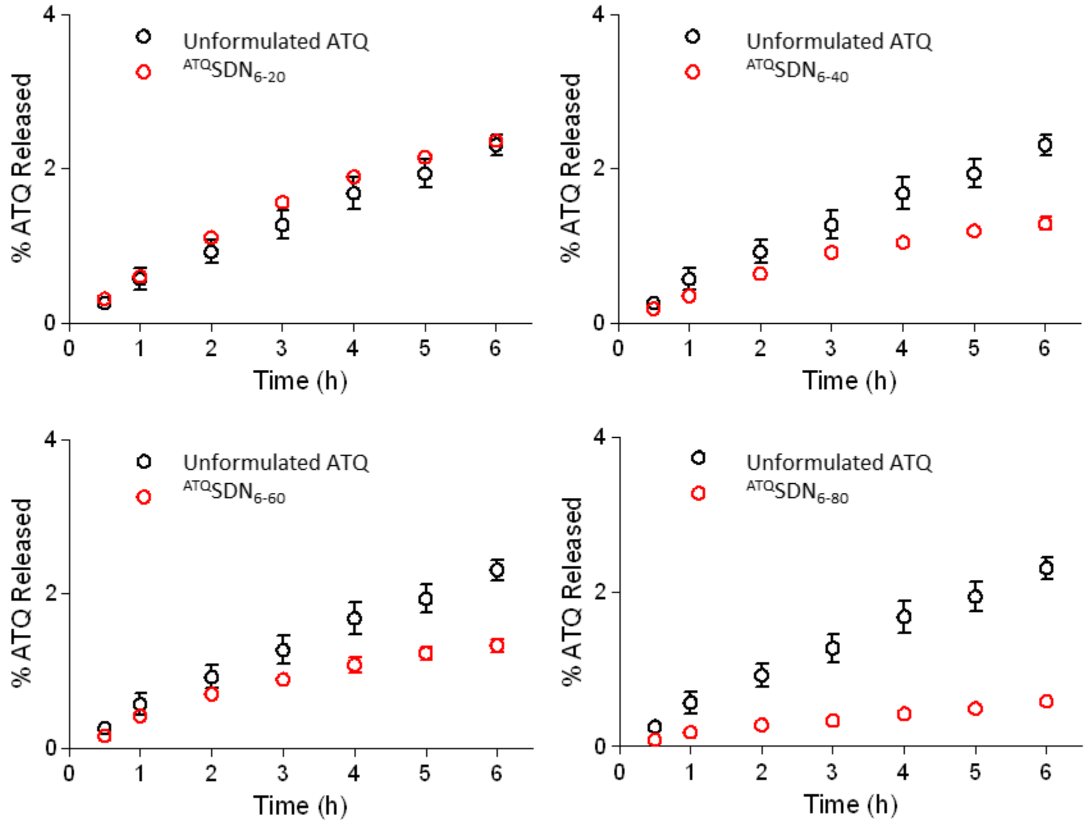

Supplementary Fig. 3 Comparison of rapid equilibrium dialysis release rate measurements of $^{ATQ}SDN_6$ at 20 ($^{ATQ}SDN_{6\text{-}20}$), 40 ($^{ATQ}SDN_{6\text{-}40}$), 60 ($^{ATQ}SDN_{6\text{-}60}$) and 80 ($^{ATQ}SDN_{6\text{-}80}$) wt% drug loading. For release rate studies, samples were prepared incorporating ^{3}H-atovaquone at 0.2 µCi mg^{-1} specific activity. $^{ATQ}SDN_6$ was selected on the basis that it exhibited the lowest release rate in the initial screen.

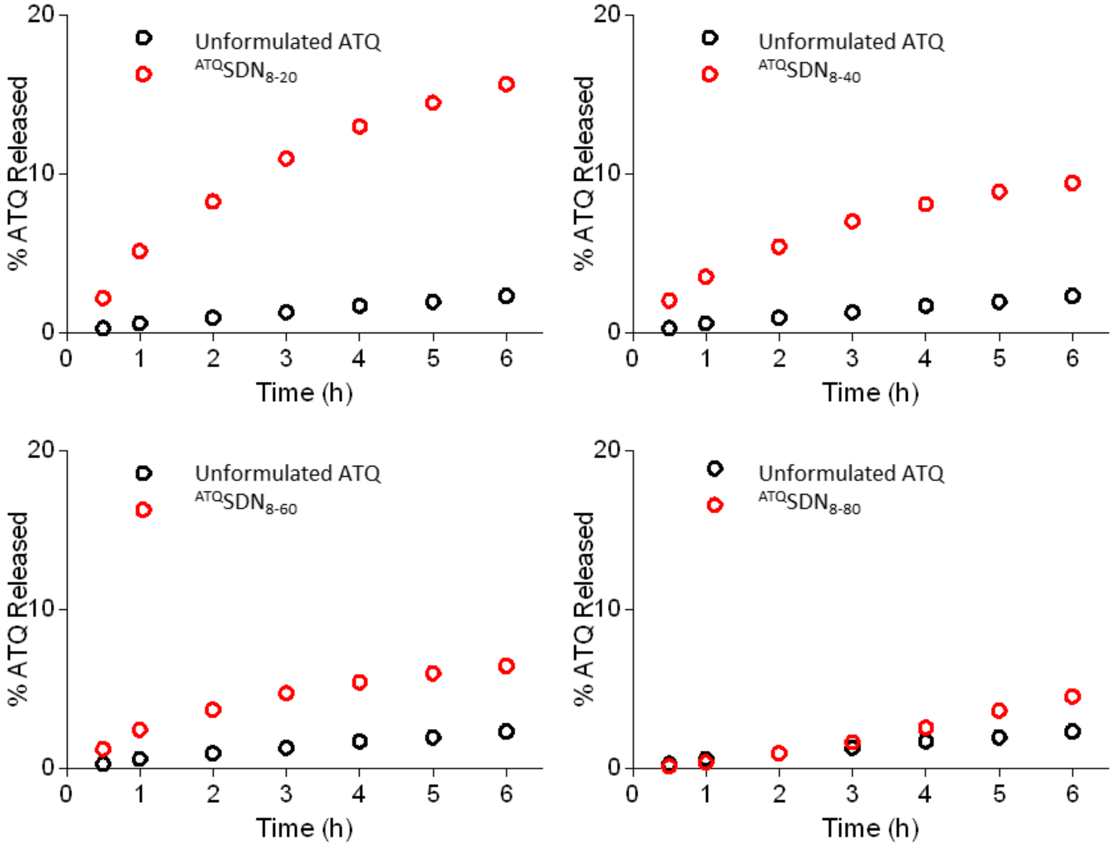

Supplementary Fig. 4 Comparison of rapid equilibrium dialysis release rate measurements of $^{ATQ}SDN_8$ at 20 ($^{ATQ}SDN_{8\text{-}20}$), 40 ($^{ATQ}SDN_{8\text{-}40}$), 60 ($^{ATQ}SDN_{8\text{-}60}$) and 80 ($^{ATQ}SDN_{8\text{-}80}$) wt% drug loading. For release rate studies, samples were prepared incorporating ^{3}H-atovaquone at 0.2 µ Ci mg^{-1} specific activity. $^{ATQ}SDN_8$ was selected on the basis that it exhibited an intermediate release rate in the initial screen.

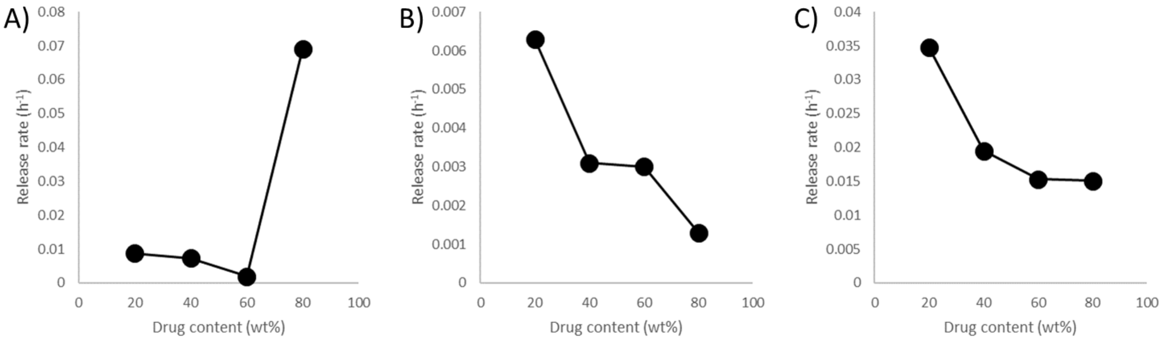

Supplementary Fig. 5 Rapid equilibrium dialysis release rates of ^{ATQ}SDN nanoformulations with systematically varying atovaquone loading relative to excipients at 6 hours. $^{ATQ}SDN_4$ (A), $^{ATQ}SDN_6$ (B) and $^{ATQ}SDN_8$ (C) were selected on the basis that they exhibited the highest, lowest and intermediate release rate during screening, respectively.

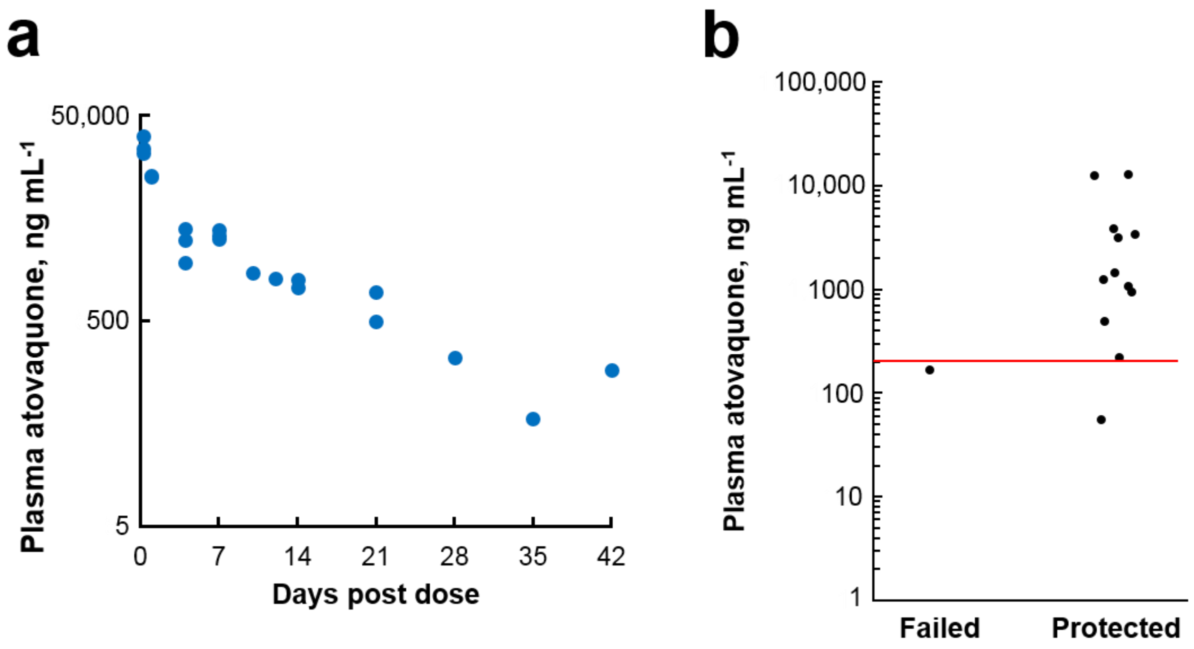

Supplementary Fig. 6 Biological testing of $^{ATQ}SDN_8$. **a,** Plasma was collected at indicated intervals for assay of atovaquone concentrations in mice dosed intramuscularly with 200 mg kg^{-1} $^{ATQ}SDN_8$. Log-transformed concentrations yield a plasma half-life of 163 h (using 4 - 42 d values, inclusive; R^2, 0.889); data obtained in 4 independent experiments. **b,** Plasma atovaquone concentrations >200 ng mL^{-1} at the time of challenge correlate closely with efficacy. Each dot represents a cohort of 3-5 mice, 7 to 42 d after a single intramuscular dose of 50, 100 or 200 mg kg^{-1} $^{ATQ}SDN_8$. Data from four independent experiments.

Supplementary Tables

Supplementary Table 1: Nanoformulation 'hits' from synthesis screen. All contain 80 wt% atovaquone.

**Formulation**	**Polymer** (13% by weight)	**Surfactant** (7% by weight)	$\boldsymbol{D_z}^{a}$ (nm)	**PdI**[b]	**Zeta Potential** (mV)
$^{ATQ}SDN_1$	Kollicoat	TPGS	477	0.281	8.2
$^{ATQ}SDN_2$	PVP K30	TPGS	346	0.261	-19.5
$^{ATQ}SDN_3$	Kollicoat	Tween 20	440	0.322	-16.3
$^{ATQ}SDN_4$	PVP K30	Tween 20	388	0.284	-16.4
$^{ATQ}SDN_5$	PVA	Tween 80	526	0.369	-20.4
$^{ATQ}SDN_6$	Kollicoat	Tween 80	454	0.309	-12.8
$^{ATQ}SDN_7$	PVP K30	Tween 80	298	0.296	-16.4
$^{ATQ}SDN_8$	PVA	NDC	445	0.345	-12.2
$^{ATQ}SDN_9$	PVA	Solutol	517	0.352	-15.5
$^{ATQ}SDN_{10}$	Kollicoat	Solutol	440	0.332	-17.0
$^{ATQ}SDN_{11}$	PVP K30	Solutol	384	0.337	-17.2

[a] D_z = Z-average diameter; [b] PdI = Polydispersity index

Supplementary Table 2: Physical characterisation of ^{ATQ}SDN nanoformulations with systematically varying drug loading relative to excipients. For release rate studies, samples were prepared incorporating ^{3}H-atovaquone at 0.2 µCi mg^{-1} specific activity.

Sample	ATQ wt%	Name	wt%	Name	wt%	D_z (nm)[a]	PdI[b]
$^{ATQ}SDN_{4-20}$	20	PVP K30	52	Tween 20	28	505	0.151
$^{ATQ}SDN_{4-40}$	40	PVP K30	39	Tween 20	21	407	0.222
$^{ATQ}SDN_{4-60}$	60	PVP K30	26	Tween 20	14	439	0.322
$^{ATQ}SDN_{6-20}$	20	Kollicoat	52	Tween 80	28	669	0.226
$^{ATQ}SDN_{6-40}$	40	Kollicoat	39	Tween 80	21	468	0.236
$^{ATQ}SDN_{6-60}$	60	Kollicoat	26	Tween 80	14	510	0.302
$^{ATQ}SDN_{8-20}$	20	PVA	52	NDC	28	891	0.199
$^{ATQ}SDN_{8-40}$	40	PVA	39	NDC	21	524	0.322
$^{ATQ}SDN_{8-60}$	60	PVA	26	NDC	14	438	0.256

[a] D_z = Z-average diameter; [b] PdI = Polydispersity index
